# Altered thrombin generation with prothrombin complex concentrate is not detected by viscoelastic testing: an *in vitro* study

**DOI:** 10.1016/j.bja.2024.10.047

**Published:** 2025-01-04

**Authors:** Nikolaus Hofmann, Herbert Schöchl, Johannes Zipperle, Johannes Gratz, Felix C.F. Schmitt, Daniel Oberladstätter

**Affiliations:** 1 Medical University of Vienna, Department of Anaesthesia, Intensive Care Medicine and Pain Medicine, Division of General Anaesthesia and Intensive Care Medicine, Vienna, Austria; 2 Ludwig Boltzmann Institute for Traumatology, The Research Center in Cooperation with AUVA, Vienna, Austria; 3 Department of Anesthesiology and Intensive Care Medicine AUVA Trauma Center Salzburg, Academic Teaching Hospital of the Paracelsus Medical University, Salzburg, Austria; 4 Department of Anesthesiology, Heidelberg University Hospital, Heidelberg, Germany

**Keywords:** point-of-care coagulation testing, prothrombin complex concentrate, thrombin generation, trauma-induced coagulopathy, viscoelastic testing

## Abstract

**Background:**

Bleeding guidelines currently recommend use of viscoelastic testing (VET) to direct haemostatic resuscitation in severe haemorrhage. However, VET-derived parameters of clot initiation, such as clotting time (CT) and activated clotting time (ACT), might not adequately reflect a clinically relevant interaction of procoagulant and anticoagulant activity, as revealed by thrombin generation assays. The aim of this study was to evaluate the ability of CT and ACT to indicate thrombin generation activity.

**Methods:**

Citrated whole blood obtained from 13 healthy volunteers underwent a 50% crystalloid dilution (DL-50%), followed by spiking with four-factor prothrombin complex concentrate (DL-50% + 4F-PCC). Changes in thrombin generation activity were compared with the VET parameters CT and ACT derived from four commercially available viscoelastic devices (ROTEM® Delta, ClotPro®, TEG®6s, and Quantra®) and standard coagulation tests.

**Results:**

Dilution of whole blood resulted in a marked increase in velocity index, peak height, and endogenous thrombin potential (all *P*<0.01), with a further substantial increase after spiking with 4F-PCC (all *P*<0.001). In contrast, CT and ACT were significantly prolonged in response to DL-50% on all devices (all *P*<0.05). Subsequent spiking of diluted blood with 4F-PCC had no impact on CT and ACT derived from VET analysers, but it restored standard coagulation tests without reaching baseline values (all *P*<0.01).

**Conclusions:**

Upregulated thrombin generation parameters after PCC spiking were not displayed by CT, ACT, or standard tests. Our results do not support treatment algorithms using prolonged CT or ACT as a trigger for administration of PCC to augment thrombin generation.

**Editor's key points**

• The viscoelastic testing-derived parameters of clot initiation, clotting time (CT) and activated clotting time (ACT), might not reflect the clinically relevant interaction of procoagulant and anticoagulant factors as revealed by thrombin generation assays.
• The ability of CT and ACT to indicate thrombin generation activity was tested *in vitro* in whole blood obtained from 13 healthy volunteers after simulated dilutional coagulopathy with or without spiking with four-factor prothrombin complex concentrate (4F-PCC).
• Dilution of whole blood markedly increased thrombin generation owing to imbalance of procoagulant and anticoagulant factors, with a further increase after spiking with 4F-PCC, whereas CT and ACT were prolonged.
• Treatment algorithms using prolonged CT and ACT as a trigger for administration of PCC to augment thrombin generation could result in dangerous hypercoagulability, a finding that should be confirmed *in vivo.*

Coagulopathy and severe bleeding are associated with considerable morbidity and mortality.^1^ Standard coagulation tests (SCTs), such as prothrombin time (PT), international normalised ratio (INR), and activated partial thromboplastin (aPTT) time, are still used to diagnose coagulation abnormalities and serve as surrogate parameters for altered thrombin generation.^2^ Thrombin is a central enzyme in the coagulation process.^3^ It cleaves fibrinogen into fibrin monomers, and activates platelets and a variety of procoagulants and anticoagulants. Impaired thrombin generation is related to a loss of coagulation factors as a result of dilution or consumption, hypothermia, acidosis, or use of antithrombotic agents such as vitamin K antagonists and direct oral anticoagulants.^4^^,^^5^ Critically low levels of thrombin generation are associated with a clinically apparent bleeding tendency.^6^ However, SCTs halt when only 5–7% of total thrombin is generated.^7^ Moreover, SCTs do not fully display the anticoagulant counterpart, which limits their use in assessing haemostatic balance *in vivo* and questions their use for predicting bleeding.^8^^,^^9^ Studies in trauma patients and patients with liver disease have demonstrated that despite prolonged SCTs, thrombin generation activity can be upregulated.^10^^,^^11^ In contrast, prolonged PT accurately displays diminished thrombin generation in warfarin-treated patients, whereas prolonged aPTT is a sound surrogate for reduced thrombin generation in patients with haemophilia. Thus, agreement between SCTs and thrombin generation is determined by the clinical scenario and ultimately by the underlying coagulation abnormality.

Viscoelastic tests are widely used to guide haemostatic therapies and are recommended by several guidelines for bleeding management.^12^, ^13^, ^14^ Clotting time (CT; ROTEM®, ClotPro®, and Quantra®) and activated clotting time (ACT; rapid TEG) reflect the initiation phase of viscoelastic measurements. Like SCTs, CT and ACT are considered surrogates for thrombin generation and are commonly used to guide therapy directed towards augmentation of thrombin generation.^15^^,^^16^ However, CT and ACT measurements stop when a defined clot amplitude (2 mm; ROTEM®, ClotPro®, and TEG6S®) is reached.^15^ Therefore, both adequate thrombin generation and a sufficient quantity of substrate (primarily fibrinogen) are essential components for a normal initiation time in VET.^16^ After the correction of hypofibrinogenaemia in bleeding patients, current guidelines recommend the use of CT and ACT to guide administration of prothrombin complex concentrate.^12^

Thrombin generation assays have contributed substantially to elucidating mechanisms of poorly understood complex coagulation disorders, such as in chronic liver disease, but they are not yet in routine clinical use.^17^ After extrinsic activation using tissue factor and phospholipids, net thrombin activity can be obtained in either platelet-poor plasma or platelet-rich plasma under the opposing action of procoagulant and anticoagulant drivers.^18^ However, thrombin generation assays are limited in their point-of-care applicability owing to intricate sample preparation, a lack of standardisation among assays, and long turnaround times.^18^

In this *in vitro* study, we investigated the effect of a 50% dilution of whole blood followed by four-factor prothrombin complex concentrate (4F-PCC) spiking on thrombin generation parameters, CT and ACT derived by four commercially available VET devices, and SCTs. The hypothesis was that CT and ACT, which are often used as surrogate parameters to detect and augment thrombin generation, do not correspond to the endogenous thrombin potential (ETP), a parameter reflective of thrombin generation capacity.

## Methods

This prospective single-centre study was approved by the local Ethics Committee (approval number AUVA EK 06/2020) and conducted at the Ludwig Boltzmann Institute for Traumatology in Vienna, Austria. After obtaining written informed consent, blood samples were collected from 13 healthy volunteers. All participants were >18 yr of age, did not take antiplatelet or anticoagulation medication, and had no known coagulation disorder or history of thromboembolism.

For viscoelastic measurements, 50 ml of blood was drawn in five 10-ml tubes containing buffered trisodium citrate 3.2% (S-Monovette Coagulation; Sarstedt AG, Numbrecht, Germany), corresponding to a citrate-to-blood ratio of 1:9. One additional 1.6-ml tube of EDTA blood was collected for a blood cell count (S-Monovette Haematology; Sarstedt AG). All samples were processed within 2 h after blood withdrawal.

The five 10-ml tubes were then pooled in a 50-ml container, split into three groups and labelled: (i) whole blood (WB): 12 ml citrated whole blood; (ii) 50% dilution (DL-50%): 6 ml citrated whole blood diluted with 6 ml isotonic saline (0.9% NaCl; Fresenius, Braun, Melsungen, Germany); and (iii) DL-50% + PCC: 6 ml citrated whole blood spiked with 5.625 ml saline and 375 μl 4F-PCC (Cofact®; Sanquin Plasma Products B.V., Amsterdam, The Netherlands). PCC spiking was performed after whole blood was diluted 1:1 with saline, yielding a concentration of ∼0.78 IU ml^−1^. The amount of 375 μl of 4F-PCC suspended in a volume of 12 ml was calculated to correspond to a dose of 3500 IU ml^−1^ (50 IU kg^−1^ BW) for an adult patient with a total blood volume of 4.5 L. In contrast to other 4F-PCC products, Cofact® does not contain heparin.

After measurement, citrated blood samples were centrifuged at 2500×*g* for 10 min to obtain platelet-free plasma, which was stored at –80°C for further thrombin generation analysis.

### Thrombin generation

Thrombin generation in platelet-free plasma was assessed using a quantitative fluorogenic assay (STG®-BleedScreen; Stago, Asnières-sur-Seine, France) on an in vitro diagnostic--certified thrombin generation analyser (ST Genesia®; Stago). Frozen citrated plasma samples were thawed at room temperature and kept on ice before analysis. The device triggers coagulation using thrombin generation and phospholipids and carries out a standardised measurement of the catalytic activity of the generated thrombin over time using an artificial fluorogenic substrate. The following parameters were obtained from the measurement: lag time (1.9–4.4 min), time to peak (4.2–9.0 min), peak height (92–320 nm), ETP (706–1767 nm∗min), and velocity index (26–132 nm min^−1^). Normal reference ranges for the measured thrombin generation parameters have been published.^19^

### Viscoelastic testing devices

Four commercially available viscoelastic analysers were investigated. Quality controls for all devices were performed according to the manufacturer's instructions.

#### ROTEM® Delta

ROTEM® Delta (Werfen, Barcelona, Spain) is a four-channel viscoelastic analyser that uses a mechanical cup and pin system. For this study, the extrinsically activated test applying recombinant tissue factor CT (EXTEM; Tem Innovations GmbH, Munich, Germany; reference range 43–82 s) was analysed.^16^

#### ClotPro®

ClotPro® (Haemonetics, Braintree, MA, USA) is a six-channel device that applies rotational thromboelastometry principles, similar to ROTEM**®**. For the extrinsically activated CT (EX-test; enicor GmbH, Munich, Germany; reference range 38–65 s), recombinant tissue factor is used.^16^

#### TEG6s®

TEG6s® (Haemonetics) is a cartridge-based VET device. A blood sample is exposed to vibrations of frequencies between 20 and 500 Hz, and clot strength is assessed by measuring the resonance frequency. We used a citrated rapid TEG (CRT), which activates the blood sample extrinsically via tissue factor and intrinsically via kaolin, and measured the ACT (reference range 82–152 s).^20^

#### Quantra®

Quantra® (HemoSonics, LLC, Charlottesville, VA, USA) is also a cartridge-based device that applies high-frequency ultrasound pulses using Sonic Estimation of Elasticity via Resonance (SEER) technology. Sonorhaeometry measures clot stiffness in hecto Pascals (hPA). We used the QStat cartridge (KT-0022; HemoSonics, LLC), which reports CT (reference range 113–164 s) after intrinsic activation using kaolin.^21^

### Standard coagulation tests

SCTs were measured in platelet-poor plasma after centrifugation at 2500×*g* for 10 min on STA Compact Max 3® (Stago) device. The PT (reference range 11.0–16.1 s; STA-NeoPTimal; Stago), aPTT (reference range 24–35 s; Cephascreen; Stago), fibrinogen (reference range 200–400 mg dl^−1^; STA-Liquid Fib; Stago), and antithrombin III (ATIII; reference range 18–120%; STA-Stachrom ATIII; Stago) were measured for each group.

### Statistics

This study was designed as a pilot; therefore, no formal sample size calculation was performed. We chose the number of study participants according to our previous experience with similar experimental studies and based on Julious's^22^ recommendations for pilot studies.^23^ Data distribution was evaluated using the D'Agostino–Pearson test. If not indicated otherwise, data are presented as mean (standard deviation). For normally distributed data, repeated-measures one-way analysis of variance with Tukey's *post hoc* test were applied, and for non-normally distributed data, the Friedmann test with Dunn's multiple comparisons test was used. To evaluate the correlation between the parameters of thrombin generation and CT and ACT, Pearson's correlation coefficient or Spearman's rank correlation was calculated.

A *P*-value <0.05 was considered statistically significant. Statistical analysis and graphical representation were performed using Prism version 10 (GraphPad Software, San Diego, CA, USA).

## Results

Blood samples were drawn from six male and seven female volunteers, with a mean age of 49 (range 24–64) yr.

### Coagulation profile of fresh whole blood

**Fig 1:**
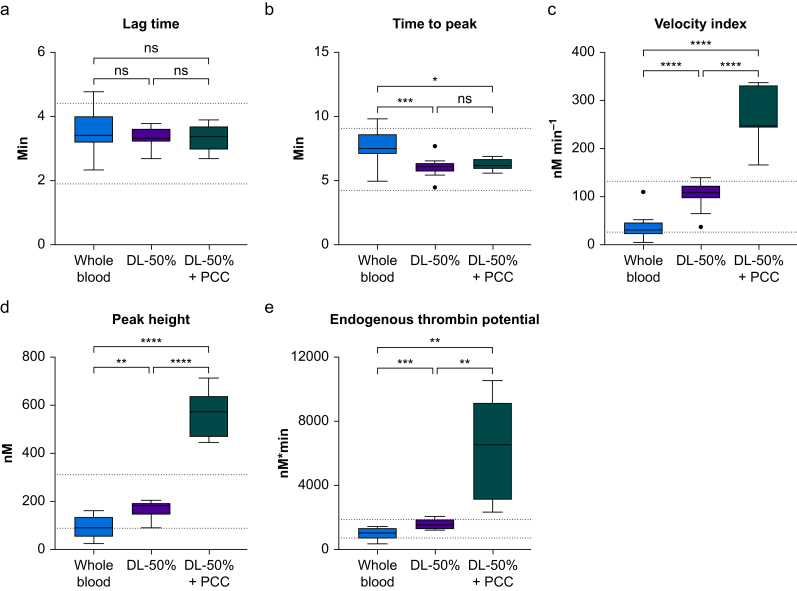
Parameters of thrombin generation measured in whole blood, 50% dilution of whole blood (DL-50%), and 50% dilution of whole blood spiked with prothrombin complex concentrate (DL-50% + PCC). Data are presented as box plots and whiskers using Tukey's *post hoc* test. Single dots represent outliers. Dotted lines denote reference ranges. ns, not significant. ∗*P*<0.05, ∗∗*P*<0.01, ∗∗∗*P*<0.001; ∗∗∗∗*P*<0.0001.

**Fig 2:**
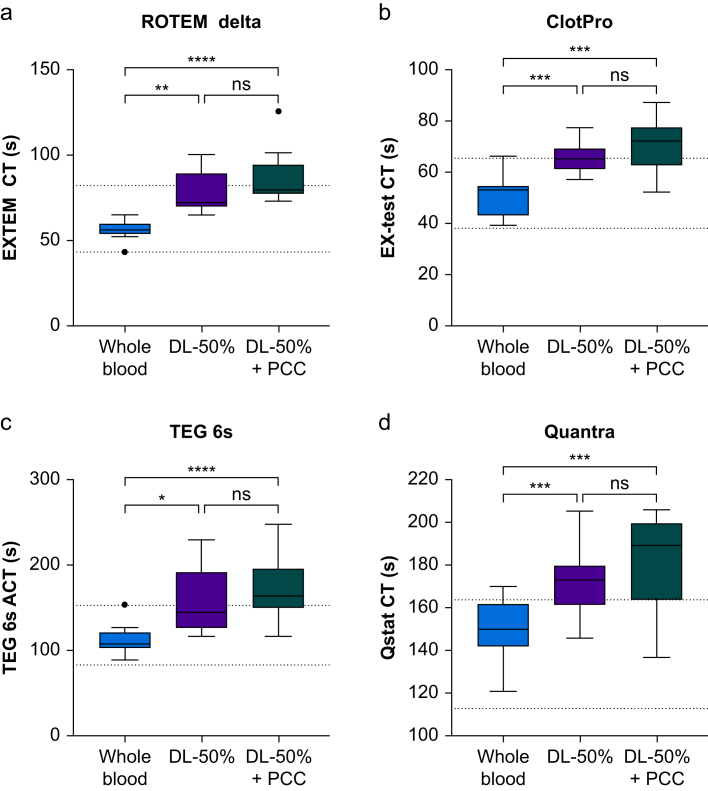
Viscoelastic parameters of clot initiation. ACT, activated clotting time; CT, clotting time; DL-50%, dilution; DL-50% + PCC, dilution + prothrombin complex concentrate. Data are presented as box plots and whiskers using Tukey's *post hoc* test. Single dots represent outliers. Dotted lines denote reference ranges. ns, not significant. ∗*P*<0.05, ∗∗*P*<0.01, ∗∗∗*P*<0.001; ∗∗∗∗*P*<0.0001.

**Fig 3:**
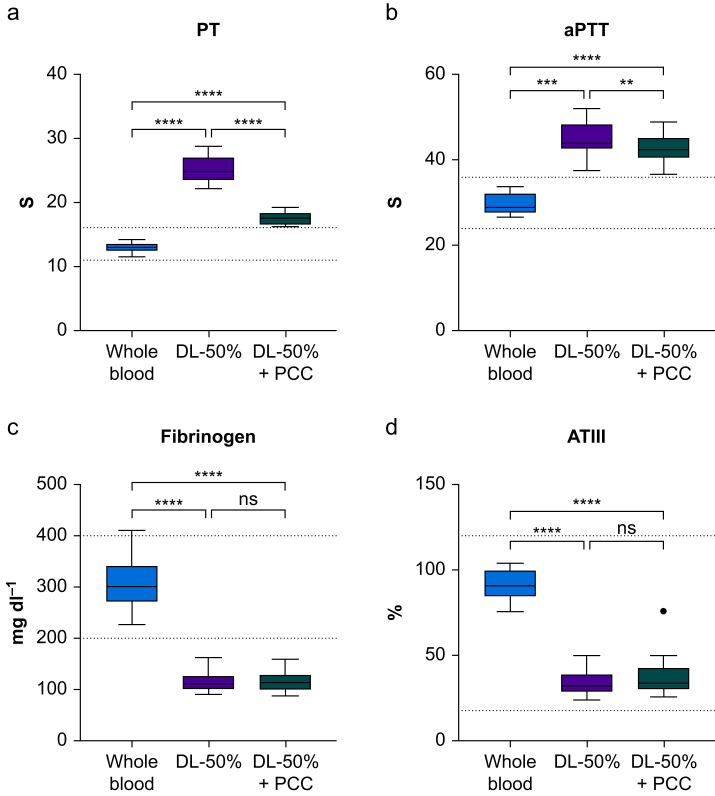
Standard coagulation tests measured in whole blood, 50% dilution of whole blood (DL-50%), and 50% dilution of whole blood spiked with prothrombin complex concentrate (DL-50% + PCC). aPTT (s), activated partial thromboplastin time; ATIII (%), antithrombin III; PT (s), prothrombin time index. Data are presented as box plots and whiskers using Tukey's *post hoc* test. Single dots represent outliers. Dotted lines denote reference ranges. ns, not significant. ∗∗*P*<0.01, ∗∗∗*P*<0.001, ∗∗∗∗*P*<0.0001.

The baseline thrombin generation levels are shown in Figure 1. CT and ACT values remained within the reference ranges provided by the manufacturers of the four investigated viscoelastic analysers (Fig. 2). Likewise, SCTs were within the normal limits of the central laboratory (Fig. 3).

### Changes in coagulation profile after 50% dilution

A 50% dilution with normal saline had no impact on the thrombin generation parameter lag time but shortened the time to peak (*P*<0.001). The quantitative thrombin generation parameters peak height, ETP, and velocity index increased (all *P*<0.01; Fig. 1). In contrast, 50% dilution prolonged CT and ACT in all investigated viscoelastic analysers (all *P*<0.01; Fig. 2). Likewise, 50% dilution prolonged PT and aPTT and decreased fibrinogen and ATIII (all *P*<0.0001; Fig. 3).

### Changes in coagulation profile after spiking with prothrombin complex concentrate

Spiking with 4F-PCC markedly increased peak height, ETP, and velocity index (all *P*<0.01). However, lag time and time to peak remained unaffected (Fig. 1). Because of fully saturated reactions and high thrombin activity beyond the upper limit of detection, the thrombin generation analyser failed to report on five samples in the DL-50% + PCC group. Hence, data could not be accessed, and these measurements were excluded from the final analysis. In contrast, spiking of the diluted blood sample with 4F-PCC had no effect on the initiation phase (CT and ACT) for any of the tested devices (Fig. 2). Although remaining below baseline levels, PT was shortened in response to 4F-PCC spiking (*P*<0.001). Similarly, aPTT decreased slightly after 4F-PCC spiking compared with DL-50%, but fibrinogen levels and ATIII were unaffected (*P*<0.01; Fig. 3).

### Correlation between thrombin generation parameters and the initiation phase of coagulation

**Fig 4:**
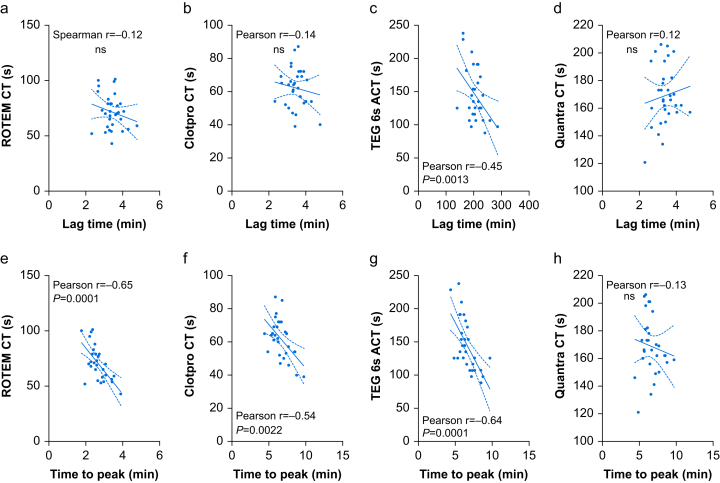
Correlations between lag time and CT and ACT (a to d) and between time to peak thrombin generation and CT and ACT (e to h). ACT, activated clotting time; CT, clotting time. Data are presented as scatterplots (a to h) showing a line of best fit and 95% confidence intervals. Either Pearson or Spearman's correlation coefficients are depicted for each graph. Significance was tested using a two-tailed test.

**Fig 5:**
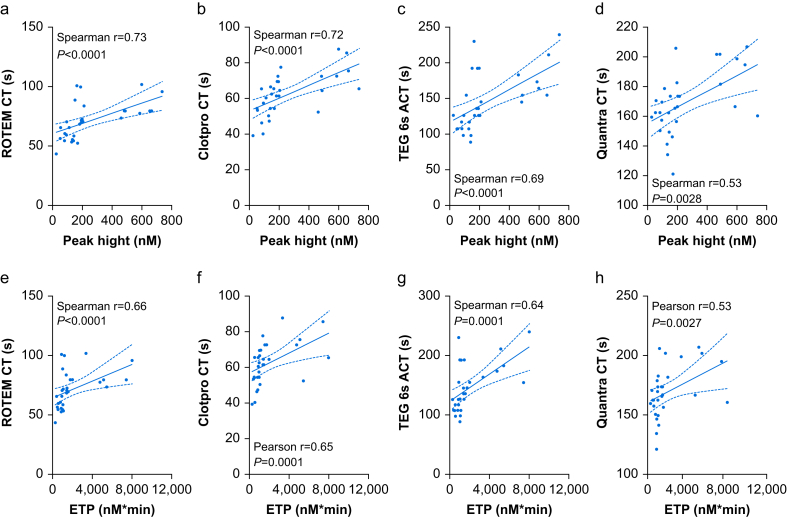
Correlations between peak hight and CT and ACT (a to d) and between ETP and CT and ACT (e to h). ACT, activated clotting time; CT, clotting time; ETP, endogenous thrombin potential. Data are presented as scatterplots (a to h) showing a line of best fit and 95% confidence intervals. Spearman's correlation coefficients are depicted for each graph. Significance was tested using a two-tailed test.

Correlation analysis revealed no relationship between lag time and CT and ACT on any of the four VET devices investigated. Similarly, the analysis revealed no positive correlation between the time to peak and CT or ACT. In contrast, strong negative correlations were observed between ROTEM® EXTEM CT and TEG6s® ACT and time to peak (Fig. 4). The quantitative thrombin generation parameters peak height and ETP showed strong positive correlations with ROTEM® EXTEM CT, ClotPro® EX-Test CT, and TEG6s® CRT ACT (all *P*<0.0001). Moderate positive correlations were detected between both, peak height and ETP, and Quantra CT (Fig. 5).

## Discussion

A 50% dilution of whole blood with normal saline resulted in upregulated thrombin generation activity compared with baseline values. Spiking with 4F-PCC yielded a further substantial increase in thrombin generation displayed by both peak height and ETP, with several samples reaching the upper limit of detection as a result of oversaturation. The most important finding of this *in vitro* study is that none of the four investigated viscoelastic analysers was able to display this increased thrombin activity through changes in CT or ACT. Furthermore, SCTs did not adequately mirror changes in thrombin activity.

As early as 2009, Dunbar and Chandler^10^ reported that trauma patients with INR >1.5 had shorter lag times and higher peak thrombin generation than uninjured subjects. Despite this well-described discrepancy, prolonged SCTs still serve as a surrogate for coagulopathy or diminished thrombin generation,^24^, ^25^, ^26^ and INR >1.2 is still used as an inclusion criterion for studies investigating trauma-induced coagulopathy.^27^, ^28^, ^29^ Our study confirms the disagreement between SCTs and changes in thrombin generation parameters *in vitro*.

We found that a 50% dilution of whole blood with a crystalloid solution upregulated thrombin generation, which shortened the time to peak and increased the peak height ETP and velocity index, while lag time remained unchanged compared with whole blood. *Ex vivo* dilution results in a more pronounced decrease in ATIII, the most important physiologic anticoagulant, compared with a decrease in procoagulants.^10^ This imbalance between procoagulants and anticoagulants increased thrombin generation up to a dilution of ∼60%.^10^ Importantly, spiking of the diluted blood sample with 4F-PCC further increased the thrombin generation parameters peak height, ETP, and velocity index. In contrast, 4F-PCC spiking had no impact on CT and ACT derived from the viscoelastic analysers.

By definition, measurement of CT and ACT stops when a clot amplitude of 2 mm is reached. Thus, normal CT and ACT VET values require both sufficient thrombin generation and adequate amounts of substrate, particularly fibrinogen. We found no meaningful correlation between CT, ACT, and lag time in response to dilution or 4F-PCC spiking. Of note, fibrinogen levels decreased by about one-third compared with baseline values after 50% dilution. Therefore, the substrate was too low in all groups to form the required initial clot amplitude of 2 mm in a reasonable time. This is why current European trauma bleeding guidelines recommend PCC supplementation to normalise CT and ACT only when fibrinogen levels are within normal limits.^12^ In contrast, anticoagulation with warfarin revealed an excellent correlation between EXTEM CT and lag time.^30^ This might be related to the fact that there is typically sufficient fibrinogen available in anticoagulated patients, meaning that the initiation phase of the coagulation process is primarily dependent on thrombin generation.

Data obtained from both *in vitro* experiments and clinical studies indicate the key role of fibrinogen in the initiation time (CT, ACT) of clot formation by VET. In a dilutional study assessing thrombin generation after use of five different PCCs, Grottke and colleagues^31^ found that EXTEM CT was restored to baseline only in a subset of samples spiked with additional fibrinogen. However, none of the tested PCCs showed an effect on EXTEM CT. Gratz and colleagues^23^ demonstrated in an *in vitro* study that despite extreme haemodilution, substitution with fibrinogen concentrate shortened EXTEM CT, whereas 4F-PCC had no effect on this parameter. In an *in vitro* study comparing two PCC preparations at different dilutional levels, Infanger and colleagues^32^ demonstrated that high thrombin activity was not adequately reflected by ROTEM EXTEM CT or ClotPro EX-test CT. In contrast to the present study, EXTEM CT was shortened in both 4F-PCC groups at all dilutional levels. Importantly, all groups were spiked with fibrinogen concentrate before 4F-PCC spiking, which is a plausible explanation for the considerable differences compared with our study. Furthermore, in a retrospective analysis of severely traumatised bleeding patients with ROTEM measurements before and after treatment with fibrinogen concentrate, 4F-PCC, or a combination of both, EXTEM CT was shortened only when fibrinogen concentrate was administered and not after 4F-PCC treatment.^33^

Many VET treatment algorithms and current guidelines for bleeding patients recommend administration of PCC based on prolonged CT and ACT.^12^^,^^13^^,^^27^^,^^34^ However, it appears that therapeutic interventions that focus on augmentation of thrombin generation by PCC do not fully address the complexity of CT and ACT regarding thrombin activity and the available substrate. Strongly upregulated ETP after PCC treatment has been reported in both animal models and clinical studies.^35^^,^^36^ The PROCOAG trial, which used INR >1.2 as an inclusion criterion, raised safety concerns owing to a higher rate of thromboembolic events in the 4F-PCC arm compared with standard of care, while mortality and allogenic blood transfusion remained similar compared with placebo.^28^ In a subset of 24 patients in the PROCOAG trial, thrombin generation showed comparable levels of ETP between placebo and 4F-PCC groups at baseline. However, 4F-PCC administration substantially elevated ETP 6 h after resuscitation compared with placebo.^37^ These data raise concerns regarding a prothrombotic state after PCC treatment during trauma resuscitation that might last for several days^38^ and potentially provoke thromboembolic events.^39^ Therefore, safe administration of PCC to treat hypocoagulability in bleeding patients demands accurate diagnostic guidance. Against this background, our findings strongly question the concept of guiding administration of PCC using CT and ACT.

It is important to note that essential features of the pathophysiology in coagulation, such as tissue injury, shock, and endothelial damage, were not taken into account in this *in vitro* study. *In vitro* haemodilution cannot mimic *in vivo* changes after bleeding and subsequent resuscitation. Importantly, because of the lack of blood flow in static conditions, one might overestimate the impact of fibrinogen replacement *in vitro*, as pulsatile flow continuously sweeps enzymes such as thrombin downstream. Therefore, local thrombin activity might be lower *in vivo*. Furthermore, this study did not provide measurements of serial dilution but only used a single haemodilution concentration of 50%. As CT and ACT remained within their reference ranges at 50% dilution, in a clinical scenario treatment with 4F-PCC would be unlikely. In combination with the sample size, our results cannot be directly translated into routine clinical practice.

Secondly, a direct comparison of different VET devices is hampered by their inherently different technologies and reagents.^16^^,^^20^ Pathway activation is the main determinant of VET. Although ROTEM EXTEM and ClotPro EX-test assays activate only via tissue factor, TEG6s CRT uses dual activation via tissue factor and kaolin, whereas Quantra's Qstat cartridge triggers coagulation intrinsically via kaolin. Conversely, this can also be viewed as a strength of our study showing that, despite their different technologies, none of these commercially available VET devices were able to report changes in thrombin generation.

Thirdly, the milieu in which thrombin generation is measured might have impacted the results. A recent study of trauma patients demonstrated that thrombin generation assessed in whole blood revealed different results compared with commonly used platelet-poor plasma.^40^ In the present study, we measured thrombin generation in platelet-free plasma, whereas VET was performed from whole blood. Consequently, we cannot exclude the possibility that thrombin generation measurements from whole blood might have yielded different results. Furthermore, direct comparison of VET results obtained from whole blood with thrombin generation measurements in platelet-free plasma is hampered by the lack of platelet contribution in thrombin generation measurements.

### Conclusions

We showed that substantially elevated levels of thrombin generation after 50% haemodilution and 4F-PCC spiking had no impact on CT or ACT measured on different VET devices. The concept of VET-derived CT and ACT for guidance of PCC administration is not supported by these findings *in vitro*. Hence, current guidelines for goal-directed haemostatic resuscitation should critically question the use of CT and ACT in clinical decision-making. Further studies are warranted to confirm these findings *in vivo* and to direct future research towards rapid accessibility of thrombin generation or adequate surrogate parameters at the bedside.

## Authors’ contributions

Conception and design: all authors

Data collection: DO, HS

Data analysis: NH, JZ, FS, HS, DO

Drafting the manuscript: NH, JZ, JG, FS, HS, DO

Read and agreed to the published version of the manuscript: all authors

## Funding

This study was funded through a dedicated research grant from the Medical Directorate (HMD, Forschungskonto) of the Austrian Worker's Compensation Board (AUVA) and by institutional funds from the Ludwig Boltzmann Institute for Traumatology, the research center in cooperation with AUVA, Vienna, Austria.

## Declaration of interests

HS received honoraria for participation in advisory board meetings for Bayer Healthcare, Böhringer Ingelheim, and TEM international and study grants from CSL Behring. JG has received honoraria, research funding, and travel reimbursement from Alexion, AstraZeneca, Boehringer Ingelheim, CSL Behring, Instrumentation Laboratory, Johnson & Johnson, Mitsubishi Tanabe Pharma, Octapharma, Portola, and Takeda. FS has received consulting fees from CSL Behring, Roche Diabetes and payment or honoraria for lectures, travel expenses, and scientific support from AstraZeneca, CSL Behring, enicor, and LFB. The other authors declare no conflict of interest.
